# Validation of a Natural Language Processing Algorithm for Detecting Infectious Disease Symptoms in Primary Care Electronic Medical Records in Singapore

**DOI:** 10.2196/medinform.8204

**Published:** 2018-06-11

**Authors:** Antony Hardjojo, Arunan Gunachandran, Long Pang, Mohammed Ridzwan Bin Abdullah, Win Wah, Joash Wen Chen Chong, Ee Hui Goh, Sok Huang Teo, Gilbert Lim, Mong Li Lee, Wynne Hsu, Vernon Lee, Mark I-Cheng Chen, Franco Wong, Jonathan Siung King Phang

**Affiliations:** ^1^ Saw Swee Hock School of Public Health National University Health System National University of Singapore Singapore Singapore; ^2^ National Healthcare Group Polyclinics Singapore Singapore; ^3^ School of Computing National University of Singapore Singapore Singapore; ^4^ National Centre for Infectious Diseases Singapore Singapore; ^5^ National University Polyclinics Singapore Singapore

**Keywords:** natural language processing, communicable diseases, epidemiology, surveillance, syndromic surveillance, electronic health records

## Abstract

**Background:**

Free-text clinical records provide a source of information that complements traditional disease surveillance. To electronically harness these records, they need to be transformed into codified fields by natural language processing algorithms.

**Objective:**

The aim of this study was to develop, train, and validate Clinical History Extractor for Syndromic Surveillance (CHESS), an natural language processing algorithm to extract clinical information from free-text primary care records.

**Methods:**

CHESS is a keyword-based natural language processing algorithm to extract 48 signs and symptoms suggesting respiratory infections, gastrointestinal infections, constitutional, as well as other signs and symptoms potentially associated with infectious diseases. The algorithm also captured the assertion status (affirmed, negated, or suspected) and symptom duration.

Electronic medical records from the National Healthcare Group Polyclinics, a major public sector primary care provider in Singapore, were randomly extracted and manually reviewed by 2 human reviewers, with a third reviewer as the adjudicator. The algorithm was evaluated based on 1680 notes against the human-coded result as the reference standard, with half of the data used for training and the other half for validation.

**Results:**

The symptoms most commonly present within the 1680 clinical records at the episode level were those typically present in respiratory infections such as cough (744/7703, 9.66%), sore throat (591/7703, 7.67%), rhinorrhea (552/7703, 7.17%), and fever (928/7703, 12.04%). At the episode level, CHESS had an overall performance of 96.7% precision and 97.6% recall on the training dataset and 96.0% precision and 93.1% recall on the validation dataset. Symptoms suggesting respiratory and gastrointestinal infections were all detected with more than 90% precision and recall. CHESS correctly assigned the assertion status in 97.3%, 97.9%, and 89.8% of affirmed, negated, and suspected signs and symptoms, respectively (97.6% overall accuracy). Symptom episode duration was correctly identified in 81.2% of records with known duration status.

**Conclusions:**

We have developed an natural language processing algorithm dubbed CHESS that achieves good performance in extracting signs and symptoms from primary care free-text clinical records. In addition to the presence of symptoms, our algorithm can also accurately distinguish affirmed, negated, and suspected assertion statuses and extract symptom durations.

## Introduction

### Study Background and Rationale

The world continues to be vulnerable to the threat from infectious diseases. This includes novel emerging infections, changes in the incidence or severity of common circulating pathogens, as well as the potential use of infectious agents in bioterrorism. There is thus an interest in developing infectious disease surveillance systems that can detect outbreaks, as well as provide adequate advanced warning of possible surges in incidence or hospitalization burden so as to enlist appropriate public health response efficiently [1].

At present, surveillance of infectious diseases in Singapore, such as in many jurisdictions, is largely passive in nature. In Singapore, this occurs through a central agency, Ministry of Health, which collates information via notifications of key infectious diseases by clinicians and laboratories and also performs weekly retrospective analysis of health care data using broad diagnostic groups [2]. The existing surveillance system with its traditional reliance on physician and laboratory diagnoses and reports has several limitations that may lead to delays in the recognition and notification of an outbreak. These include a dependence on timely recognition and reporting by clinicians, challenges faced by clinicians in recognizing the unexpected presentations of novel pathogens, and delays in obtaining laboratory results for agent identification [3-5]. For novel infections, in particular, the failure to suspect a case, order a laboratory test, or in some instances the unavailability of an accurate diagnostic laboratory assay may all contribute to delays in detection. Moreover, the retrospective nature and coarse grouping of conditions by diagnoses codes with use of only simple thresholds on counts of cases can miss more subtle but important signals that take into account the spatial and contextual relationships between clusters of infectious cases and possible changes in incidence, clinical presentation, or severity, even for commonly circulating pathogens. Singapore currently has universal uptake of electronic health records among its public sector health care providers, and syndromic surveillance systems leveraging on electronic medical records (EMRs) to identify syndromes may help to overcome some of these limitations by providing surveillance data that complement our existing methods for surveillance [5,6]. By grouping symptoms identified into specific syndromes based on the presentation of the illness, we may potentially identify illness clusters that would not otherwise be suspected [7,8], particularly when leveraging off other routinely available information in electronic health records, such as demographic and geolocation data [9]. However, to capture clinical presentation as syndromes requires additional intervention. We could request that doctors remember to and comply with the burden of entering additional data alongside their clinical duties as predefined syndromes (as is currently done for monitoring of influenza-like illness [10]). However, this has several drawbacks, including a need to predefine syndromes with consequent practical limits to the number of case definitions that could be in use, the need to educate all reporting parties on the case definitions, variations in interpretations of these case definitions, and potentially poor compliance. Approaches have also been developed to map diagnoses into syndromes for surveillance [11,12], but these have in some instances been found to be inadequate to detect outbreaks on their own. For instance, Lusigna and colleagues [13] found that an ontological approach to define gastrointestinal disease using all the terms and codes was better than using International Statistical Classification of Diseases and Related Health Problems-10th revision (ICD-10) alone. Another alternative to these approaches would be to rely on natural language processing (NLP) algorithms to extract from free-text information what would be routinely documented by practicing clinicians and transform such data into codified information [8]. However, free-text clinical narratives are rife with abbreviations or shorthand forms, misspellings, synonyms, and contextual information, which poses a challenge to accurately extract clinical information [8,14]. As such, NLP algorithms need to be trained and validated to achieve optimal performance.

### Aims and Objectives of the Study

The aim of the study was to describe in detail the process of creating a rule-based NLP algorithm called Clinical History Extractor for Syndromic Surveillance (CHESS) that extracts signs and symptoms associated with infectious diseases outbreaks. We also trained and validated CHESS’s performance against a manually coded reference standard and present the results in this paper.

## Methods

### Study Setting and Algorithm Development

We developed CHESS that adopts concept extraction using a rule-based approach. The tool uses part of speech tagging, prefixes, and regular expressions and incorporates ontology and grammar-based analysis to extract signs and symptoms from free-text notes. We chose the rule-based approach as it was simpler to operate and easier to create and understand than other systems based on machine learning. We felt this would thus also be an appropriate benchmark for the development of iterations of algorithms based on machine learning, which are likely to be developed in the future. Furthermore, with a good keyword dictionary adapted to local context, this tool can be easily updated to incorporate various new features and adapted to other clinical contexts. CHESS targets 48 signs and symptoms of interest from four different syndrome categories: (1) gastrointestinal infection syndromes, (2) respiratory infection syndromes, (3) constitutional signs and symptoms typically present during infectious diseases, and (4) other signs and symptoms not belonging to the former three groups (with the full categorization displayed in Multimedia Appendix 1). The choice of symptoms were based on infectious disease diagnoses categories currently monitored in Singapore [2] and were sufficiently detailed to give a flexibility to combine symptoms to construct case definitions for detecting possible future outbreaks. The mapping of symptoms to syndromes was modeled after the Centers for Disease Control and Prevention Electronic Surveillance System for the Early Notification of Community-Based Epidemics II framework [11,15].

The process began by constructing a library of keywords associated with the signs and symptoms of interest. We downloaded the 2014AB version of United Medical Language System (UMLS) [16] and identified key medical concepts. We started with the UMLS metathesaurus as it was free to use and had a comprehensive database of over 3 million medical concepts from over 150 libraries including Systematized Nomenclature of Medicine-Clinical Terms and ICD-10-clinical modification, the latter being commonly used in Singapore. The NLP module was built with ANother Tool for Language Recognition (ANTLR), which is an open source Java-based parser generator. This has been modified to include various components as per our requirements. In the first iteration of CHESS, the tool had an overall recall value of 65.4% when tested with a random dataset, indicating that a huge number of terms went undetected (false negatives). This was attributed to shorthand forms, which were common locally and misspellings within free-text notes [14,17] that were not accounted for in the UMLS metathesaurus. To broaden the dictionary and include these terms, CHESS was trained ad-hoc with two small pilot local health care datasets made available to us for preliminary developmental work before further training and validating the process on National Healthcare Group Polyclinics (NHGP) datasets as described in this paper. Training included manual addition of possible terms based on clinical notes.

**Figure 1:**
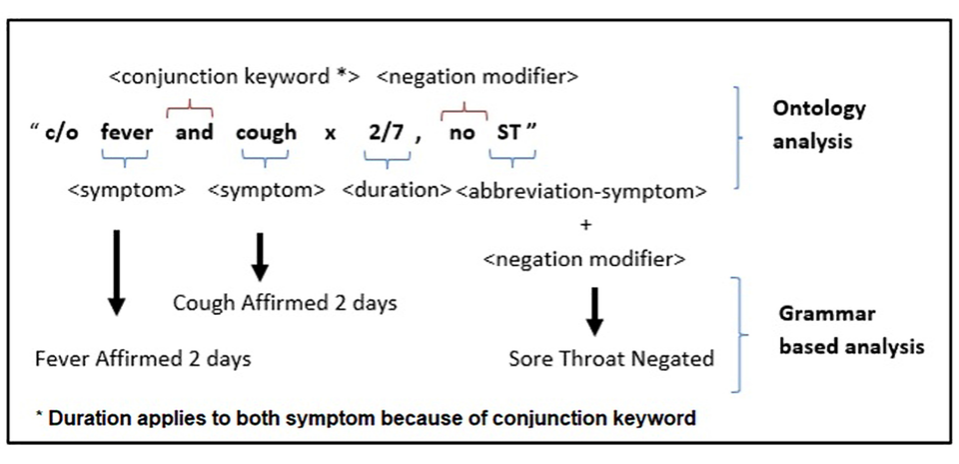
Ontology and grammar-based analysis of the rule-based natural language processing (NLP) algorithm. Signs and symptoms and information on assertion status and duration are captured and tokenized in the ontology analysis. Relationships between tokens are built up in the grammar-based analysis. C/o: complain of; ST; sore throat.

In Singapore, clinical free-text information is usually short, with each new finding separated by line breaks. In the ontology analysis, the clinical visit free text is separated into phrases by line breaks. Phrases are recursively parsed into tokens for easier categorization according to a set of lexer rules for patterns. These tokens are broadly categorized as symptoms, assertion status, and duration (Figure 1, top portion). Each symptom that is identified from the dictionary has a relational database to incorporate common misspellings, abbreviations, and synonyms. Assertion status is identified by specific terms that determined if there is a negation modifier (eg, *no*, *denies*, and *nil*). In addition, we used another set of terms indicating *suspected* status (eg, *claims* and *?<symptom>*). If these terms were present in the phrase, they will change the assertion status of symptoms in that phrase to *negated* or *suspected*, respectively. Otherwise, symptoms are identified to be *affirmed*. Negation modifiers reverse assertion status of symptom; for example *not afebrile* will be fever affirmed. Conjunction terms such as *and*, *or*, *commas* (ie*,*) and *slash* (ie*/*) are used to chain a list of signs and symptoms together in the same phrase. A stopword dictionary was built to remove nonessential words (eg, *over*, *on*, and *before*) that will interfere with exact string matches. In the grammar-based analysis phase, relationships between tokens produced in the ontology analysis are built up to make sense of the sentence (Figure 1, top portion). Patterns and grammar rules were initially built up from the UMLS and modified with inputs from domain experts. In addition, duration tokens were normalized by comparing with a duration dictionary. Temporal attributes are identified by rules that are set to associate duration to appropriate symptoms in proximity to the duration token and by taking into consideration conjunction terms. Instances where a specific onset date was given (either a calendar date or with reference to the date of consultation, for example, today and yesterday) were converted to duration terms, with onset on the day of consultation counted as 0 day and onset yesterday as 1 day. The algorithm was implemented in the ANTLR, which generates a Java implementation from a grammar file.

**Figure 2:**
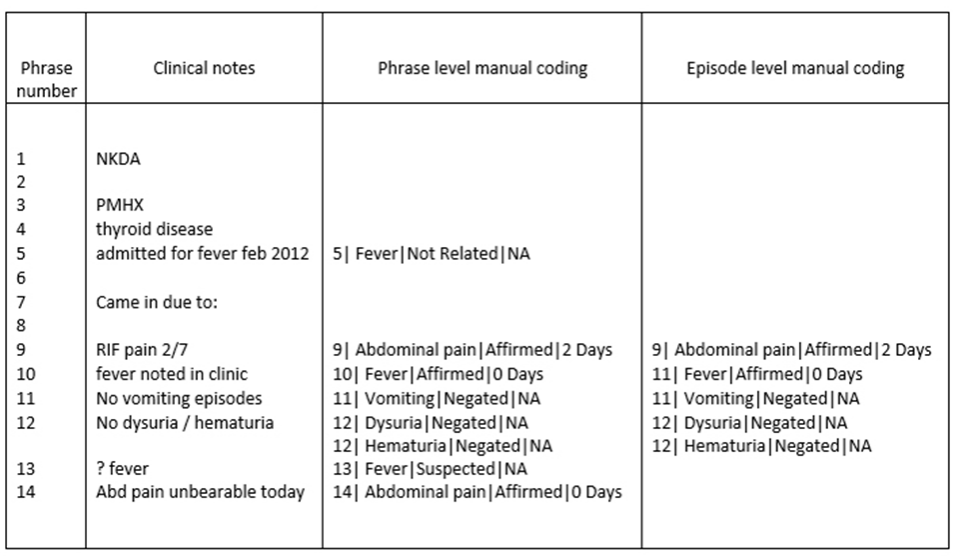
Sample set of clinical notes and transformation following phrase-level manual coding and episode-level coding. Abd: abdominal; NA: not applicable; NKDA: no known drug allergy; PMHX: past medical history; RIF: right iliac fossa.

Symptoms were then manually coded at the phrase level, and this information was used to create episode level symptom coding (Figure 2). The purpose of episode level output was to identify unique symptoms, with useful information on presence and duration from multiple entries of the same symptom in each clinical record. After phrase level symptoms were accurately identified, we utilized a set of rules to achieve episode level output. In infectious disease surveillance, the presence of a symptom (affirmed) in a patient is likely more important information than the similar symptom noted as not being present (negated) during the documentation of the same episode. Thus, the affirmation of a particular symptom was given priority over negation of that symptom recorded elsewhere in the same record. In instances where the presence of a symptom is suspected, this symptom is made void when the same symptom is negated or affirmed elsewhere in the clinical record of that episode, as we considered to this to be less certain than affirmation or negation. For symptom duration, both the manual coding and the NLP tool would identify multiple instances of symptom duration occurring at the phrase level within the same episode. Symptoms specified to have lasted for “few days” were considered to be unspecified but acute symptoms. Symptoms lasting more than 7 days, or indicated as beginning “last week” are grouped together as chronic (>1 week) symptoms. To simplify the analysis, we chose to summarize the data using the symptom with the earliest onset (ie, the longest duration) at the episode level, which we then compared against the reference standard.

### Dataset Used and Training and Validation Process

Our data was obtained from the NHGP, a major public sector chain of clinics estimated to provide about 10% of the primary care in Singapore. To facilitate batch extraction, we chose three clinics (one each from the West, North, and Central regions), then performed a stepwise random sampling of the records across the period from which EMR was available from the middle of 2009 to June 2014. For each clinic, we randomly selected 10 dates that did not fall on a Sunday or public holiday; these dates were evenly divided into 10 half-yearly periods from across the 5-year period for which the EMR was available. Subsequently, for each selected date, 56 records with at least three lines of free-text notes were randomly selected, thus giving a total of 560 records of consultation clinical records from each of the three clinics across the 5-year period. As free-text notes could potentially contain identifiable information, to comply with personal data protection regulations, every record was vetted (and where necessary redacted) by an internal staff member of NHGP before it was shared with the wider collaborative research team (including those from other institutions) for further analysis, and this process limited the total number of records that could be extracted and shared.

**Figure 3:**
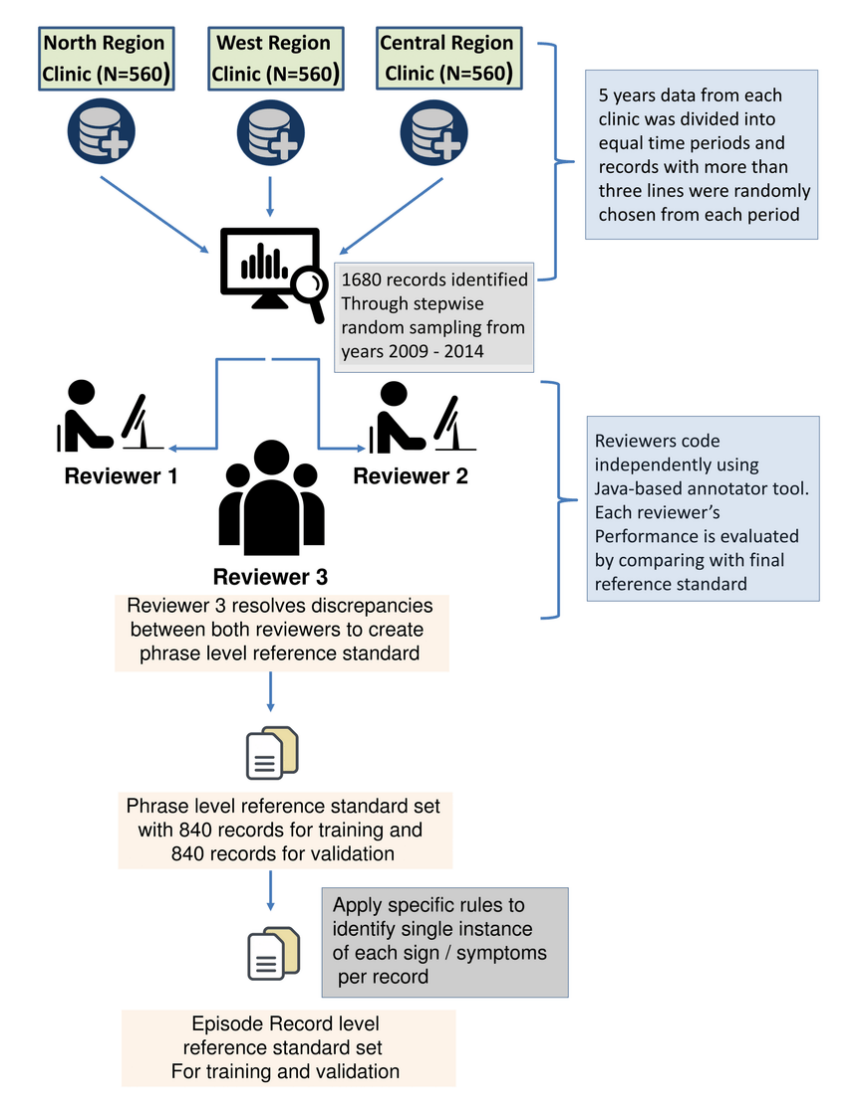
Flowchart of process for creating reference standard.

Figure 3 describes the process by which we used manual review by human coders on all extracted records to create a reference standard to train and validate CHESS’s algorithm. A Java-based annotator interface tool was created to improve manual coding methodology and prevent mistakes. Two independent human reviewers, who were health care workers with substantial experience in clinical research and interpreting medical case notes, read through the clinical records and then annotated each line of the record for the presence of signs and symptoms, the assertion status, and duration of symptoms experienced; this allowed us to capture multiple instances where a sign or symptom appeared within each record. Then a third reviewer, a clinician who has practiced in the primary care setting, served as the adjudicator in instances where the two reviewers were not in agreement.

Training of the algorithm was conducted with 840 manually annotated records to improve CHESS’s performance by identifying new terms, misspellings, and shorthand forms to be updated into the pattern and grammar library, or removing keywords that caused significant false detections. We repeated several rounds of training until we achieved satisfactory performance with the training dataset. Following training, CHESS’s performance was validated on the remaining 840 notes that were independent from the training set to test the algorithm’s robustness in correctly identifying signs and symptoms.

### Analysis of Natural Language Processing Performance Compared With Manual Coding Performance and Reference Standard

CHESS’s performance was assessed by its precision, recall, and F-measure for detecting signs and symptoms in comparison with the adjudicated reference standard. This was performed at both the phrase level and episode level. The precision, recall, and F-measure were defined by the following formulae, where true positive refers to signs and symptoms that were accurately identified by CHESS, false positive refers to signs and symptoms incorrectly identified, and false negatives refers to signs and symptoms missed:

- Precision = (True Positive) / (True Positive + False Positive)
- Recall = (True Positive) / (True Positive + False Negative)
- F–measure = (2 × [Precision × Recall]) / (Precision + Recall)

Precision is the frequency with which symptoms identified by the tool are relevant (positive predictive value). Recall is the frequency with which relevant symptoms are identified (sensitivity). We used F-measure, which is a weighted harmonic mean of precision and recall to give an overall picture of the tool’s performance. F-measure is applicable for our situation as we do not have true negatives, yet had a reference standard to compare with [18]. Again, because we do not have a true negative, we could not use Cohen Kappa statistic to review interrater reliability, and so the same metrics were also used to assess each manual coder’s performance against the final reference standard.

To assess the performance of CHESS for symptom identification, assertion status, and duration, we utilized the dictionary after it was trained with 840 records on the validation set. The performance of CHESS in capturing specific symptoms was assessed for all symptoms and also stratified by individual symptoms and visualized in a bubble chart. Performance in assigning the correct assertion status to symptoms was assessed on all true positive symptoms that were identified by both NLP and reference standard. A matrix plot was created to see where the capacity to identify assertion status was lacking.

Finally, in syndromic surveillance, an episode level onset date is helpful in characterizing the temporality of an infection, and this can be imputed if the duration of symptoms is known at the time of consultation. We present the proportion of episodes in the reference standard with a valid episode level duration (based on the earliest symptom) that were correctly identified, with additional stratification by episode duration for acute symptoms.

We also conducted a qualitative review of instances where the NLP algorithm failed to correctly identify symptoms, assertion statuses, and symptom duration and describe the potential areas for improvement.

## Results

### Description of Data Source and Common Symptoms Identified

For the 1680 primary care clinical records extracted, there were no significant differences on the genders of the patients across the North, West, and Central clinics. However, a significantly higher proportion of Chinese ethnicity (compared with Malay and Indian) and significantly older population was observed in the clinic from the North. This was in concordance with the overall population distribution within the three districts based on national demographic surveys [19]. Consequent to the older case mix, the clinic from the North also had more consultations for chronic diseases than the other two clinics.

Table 1 shows the frequencies of the 10 most commonly detected signs and symptoms from the 1680 records reviewed by human coders (full list of signs and symptoms displayed in Multimedia Appendix 1). Overall, fever was detected most frequently (12.05% [928/7703] of all instances of symptoms detection) but was in the large majority of instances “negated.” Other common signs and symptoms detected within the clinical records were those associated with upper respiratory tract infections such as cough, sore throat, rhinorrhea, and sputum, and these were in the majority of instances affirmed (between 70.38% and up to 86.82%).

### Comparison of Natural Language Processing Against Human Coders in Identifying Signs and Symptoms in Free Text

Table 2 shows that for phrase level output, both human coders have good agreement with the final adjudicated output used as the reference standard other than for a slightly lower recall for coder 2 (because of differences in interpretation of clinical examination findings and abbreviations). The final round of training led to sufficient performance, with CHESS having a precision of 95.3% and recall of 96.2% with the training set; levels which were fairly similar to those of the human clinical coders. CHESS also achieved a precision and recall of 94.2% and 90.4% with the validation set, with the lower performance because of our limitations in identifying (through the training dataset) all relevant phrase-level terms present in the validation dataset. Results for episode-level analysis (Table 3) were better, with the performance again being comparable with the human coders, with a precision and recall of 96.7% and 97.6% in the training dataset and 96.0% and 93.1% in the validation dataset, respectively.

**Table 1:** Frequency of the 10 most commonly detected signs and symptoms within 1680 primary care clinical records by human coders.

Symptoms sorted by frequency of symptom mention in episode level	All instances (N=7703), n (%)^a^	Instance of symptom affirmation, n (%)^b^
Fever	928 (12.04)	228 (24.6)
Cough	744 (9.66)	646 (86.8)
Sore throat	591 (7.67)	416 (70.4)
Rhinorrhea	552 (7.17)	435 (78.8)
Altered state of consciousness	376 (4.88)	7 (1.9)
Vomiting	347 (4.50)	75 (21.6)
Rash	345 (4.48)	72 (20.7)
Dyspnea	286 (3.71)	31 (10.8)
Diarrhea	271 (3.52)	137 (50.6)
Sputum	256 (3.32)	212 (82.8)

^a^Column percentages, with the denominator being all instances (N=7703).

^b^Row percentages, with the denominator being the instances where the symptom in that row appears (eg, for Fever, n=928).

**Table 2:** Phrase level precision, recall, and F-measure of human coders and Clinical History Extractor for Syndromic Surveillance (CHESS) outputs compared against instances of symptom occurrences in reference standard.

Performance against reference standard	Comparison of coder 1 versus coder 2 (N=8861 instances)		CHESS performance for training set (after training of dictionary, N=4282 instances)	CHESS performance for validation set (after training of dictionary, N=4578 instances)
	Coder 1	Coder 2		
Precision, %	98.52	96.06	95.24	94.15
Recall, %	96.93	84.30	96.17	90.39
F-measure, %	97.72	89.80	95.70	92.23

^a^CHESS: Clinical History Extractor for Syndromic Surveillance.

**Table 3:** Episode level precision, recall, and F-measure of human coders and Clinical History Extractor for Syndromic Surveillance (CHESS) outputs compared against instances of symptom occurrences in reference standard.

Performance against reference standard	Comparison of coder 1 versus coder 2 (N=7703 instances)		CHESS^a^ performance for training set (after training of dictionary, N=3738 instances)	CHESS performance for validation set (after training of dictionary, N=3965 instances)
	Coder 1	Coder 2		
Precision, %	98.91	97.13	96.74	95.97
Recall, %	97.46	88.47	97.65	93.06
F-measure, %	98.18	92.58	97.19	94.49

**Figure 4:**
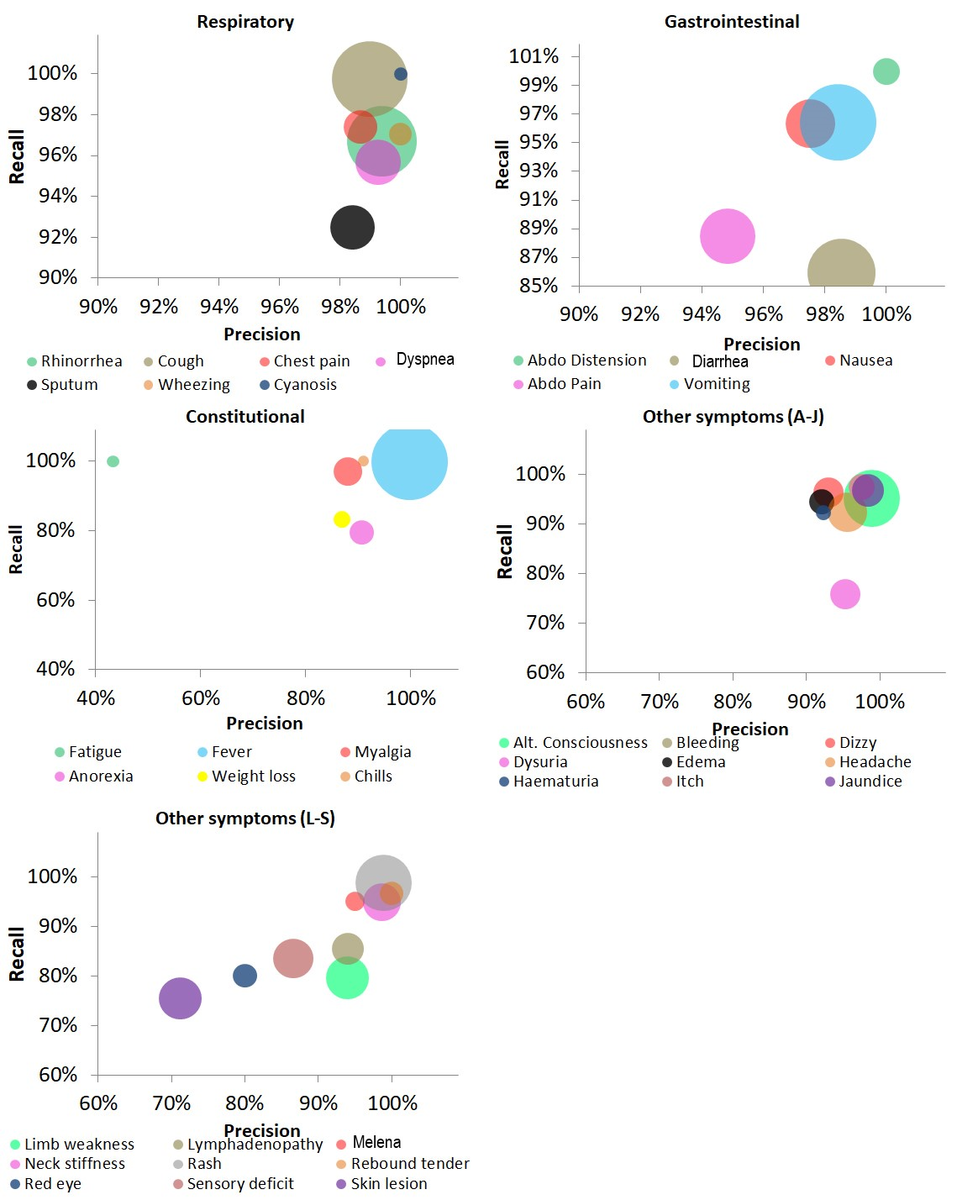
Bubble chart of the Clinical History Extractor for Syndromic Surveillance’s (CHESS’s) precision and recall for each sign and symptom in episode level analysis for the validation dataset. Each bubble denotes a single symptom categorized into symptom types: respiratory, gastrointestinal, constitutional, and others. Bubble size is proportional to the number of cases identified by humans (true positive + false negative). Symptoms present in less than 1% of records are not presented.

Figure 4 gives CHESS’s performance for specific signs and symptoms that occur in more than 1% of the medical records (see supplementary table E2 and E3 for detailed breakdown) using the validation dataset. High precision and recall of >90% were achieved for most signs and symptoms associated with respiratory and gastrointestinal syndromes. “Diarrhea” and “abdominal pain” had slightly lower recall (<90%) in the validation set, but this was limited to records where these were “negated”; recall was 97.7% and 90.6%, respectively, when diarrhea and abdominal pain was “affirmed” vs only 71.0% and 85.0% when “negated.” This was because of clinicians entering misspelled words (eg, “supropubic” pain) and new terminologies (eg, RIF) that CHESS was not able to identify resulting in high false negatives. Bleeding had the poorest recall of the symptoms with only 60.8%. This is because the word “blood” was intentionally omitted from CHESS’s list of keywords because of the generic use of the word for unrelated tests and measurements (eg, blood test and blood pressure). As such, adding “blood” into the list would have generated many false positives leading to an even worse precision for bleeding. On the other hand, fatigue was found to have a poor precision of 45.5%. This was because of the word “weakness,” also commonly used to describe limb weakness. This resulted in false positives for fatigue and false negatives for limb weakness. However, the overall recall for limb weakness was still above 80% because of the large number of true positive instances (n=175).

### Accuracy of Natural Language Processing in Identifying Assertion Status and Duration of Symptoms

**Figure 5:**
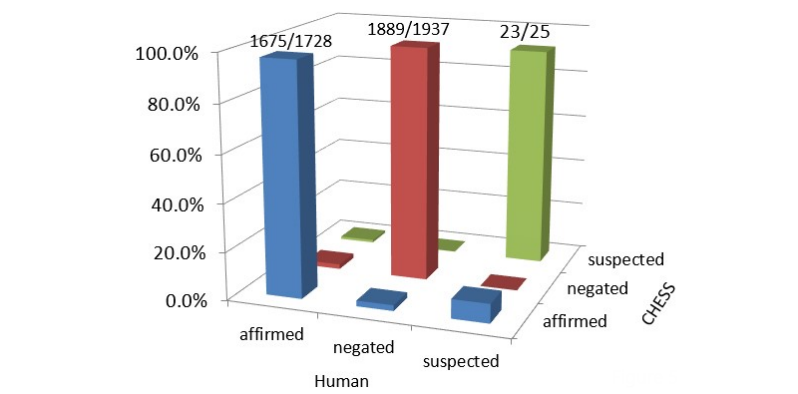
Clinical History Extractor for Syndromic Surveillance’s (CHESS’s) accuracy in identifying assertion status of symptoms within episode level analysis based on the validation dataset.

CHESS also performed well in assigning the correct assertion status to the signs and symptoms correctly identified (ie, true positives). Of 3690 instances of true positives in the validation dataset, 1728 (46.83%), 1937 (52.49%), and 25 (0.68%) were determined as affirmed, negated, and suspected, respectively, in the reference standard (Figure 5). CHESS correctly assigned the assertion status of signs and symptoms for 96.9% of instances when they were affirmed, 97.5% when they were negated, and 92.0% when they were suspected, with an overall accuracy of 97.2%. Sources of error mainly arose in three ways. First, as our tool relied on using line breaks to separate out phrases, when the whole visit was entered without any appropriate conjunction keywords in one line instead of multiple lines, the assertion status would be deemed by the NLP to apply to the all the symptoms in that line. Although this was rare given the prevailing styles of clinical text data entry, it did result in a few instances of misclassification for assertion statuses. Second, misclassification by CHESS of a symptom as affirmed occasionally occurred when doctors advised a patient of future symptoms to watch out for. Third, the keywords learned from our training dataset to identify instances where a symptom as “suspected” were not exhaustive for all the instances found in the validation dataset.

**Figure 6:**
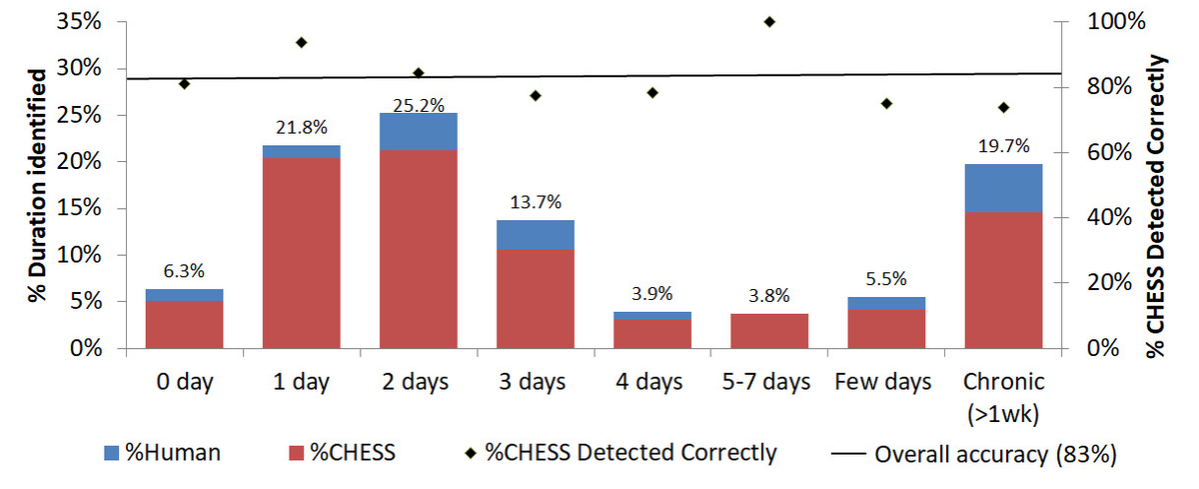
Episode level analysis on the distribution of symptom episode duration in instances detected by human coders (blue) among all the National Healthcare Group Polyclinics (NHGP) records and the distribution of durations detected by Clinical History Extractor for Syndromic Surveillance (CHESS; red) based on the validation dataset. Diamonds give the proportion of records where CHESS correctly identifies and assigns the duration information stratified by episode duration (based on the reference standard), with the horizontal line giving the aggregated accuracy for detection of symptom duration for all records analyzed.

Of 778 records with at least one sign or symptom detected in the validation dataset, 583 (75.0%) included information on the duration of the episode (Figure 6). The majority (53.3%) of these had acute onset within the past 2 days. In terms of accuracy, CHESS had an overall accuracy of 83% for detecting and assigning the correct duration. Performance was degraded largely because the rules devised based on the training dataset were not exhaustive. There were many abbreviations such as “y” for years and instances such as “2days” where the words and numbers occurred together (without an intervening space), as well as misspellings, most of which were apparent only on reviewing classification errors for the validation dataset.

## Discussion

### Principal Findings

In this work, we have described the design and performance of CHESS, a rule-based NLP algorithm that we showed is reasonably accurate in extracting information on symptoms, assertion status, and the duration of symptoms from free-text clinical notes in a set of EMR from a large primary care provider.

The performance of CHESS is comparable with results from other systems in the extant literature. For instance, a system developed by McRae et al [20] to identify influenza-like-illness from unstructured primary care notes reported a precision of 87.8% and recall of 90.2% on their validation set relative to a human reference standard. Another system, the Multi-Threaded Clinical Vocabulary Server (MCVS), was used by Matheny et al to identify specific symptoms suggestive of tuberculosis, hepatitis, and influenza from clinical notes [21]. That MCVS-based system, which can also identify assertion status, had an overall performance of 91.2% precision and 83.5% recall for detecting symptoms. The system was able to correctly identify 84.7% of positive assertions, 75.1% of negative assertions, and 0.7% of uncertain assertions. Elkin and colleagues also reported that the MCVS achieved a sensitivity of 92.9% and a specificity of 34.6% in identifying influenza infection in patients [22]. MCVS uses medical concepts from SNOMED-CT terminology, and it was noted in one study that ontology based on SNOMED-CT have a precision of 99.8% and recall of 99.7% in identifying medical problems [23].

The ability of CHESS to accurately discern whether the signs and symptoms were affirmed, negated, or suspected with very high accuracy (97.2% for validation set) is an important feature when monitoring primary care records for specific case definitions associated with particular infections. In our study, we noted that a large portion of the symptoms identified in the clinical notes were of negated status as the clinician was eliminating symptoms of the key differential diagnoses. For example, “fever” was the most frequently detected symptom, but it was far more often negated than affirmed. Failure to distinguish negation from affirmation could potentially lead to significant background noise that may mask the signal from a real outbreak.

The other key novel capability of CHESS compared with other NLP systems worth highlighting is the extraction of symptom duration in addition to the assertion status. This is particularly critical in primary care data, given that consultations for infectious disease conditions such as upper respiratory tract infections would be more common on Monday than on other days of the week and be the lowest during weekends when not all clinics are open [24]. This day-of-week variation in visitation rates potentially necessitates setting of higher daily thresholds for signaling an outbreak that again may reduce our sensitivity to detect outbreak signals [25]. Extracting symptom duration allows us to impute an estimated day of onset instead of relying only on the day of consultation. This potentially reduces day-of-week effects, with consequent improvements in temporal resolution for EMR-based surveillance.

### Application of Clinical History Extractor for Syndromic Surveillance (CHESS) to Infectious Disease Surveillance

We intentionally designed CHESS to extract individual signs and symptoms rather than predefined syndromes. Such a design facilitates the use of specific case definitions involving combinations of individual symptoms. For instance, in 2016, an outbreak of Zika virus infections in Singapore was detected when an astute primary care physician reported a cluster of patients presenting with fever, rash, and joint pains [26]. Our system would have the flexibility of including additional symptoms for a Zika virus case definition, such as conjunctivitis, which was also associated with Zika virus infections. We can also tailor case definitions to new emerging infections of concern, then monitor for unexpected clusters of such cases anywhere within the reach of our EMR systems. Other applications could include surveillance for changes in incidence or severity of commonly circulating infections of concern, such as influenza. In such an application, we could track incidence of a syndrome comprising acute onset of fever, cough (for which our algorithm performed fairly well), and a body temperature ≥38⁰C (which is a coded field in NHGP EMR) that has reasonable discriminatory value for influenza in primary care [24,27]. Combining this with hospital admissions for influenza can potentially allow us to assess age-stratified incidence and severity, which has been known to differ between influenza epidemic as well as influenza pandemic strains [28]. Applications in these areas will require further validation for specific syndromes of interest, such as by comparing disease incidence estimated from primary care data, in this case through EMR, to other independent methods [29]. Such validation work should also look into approaches that combine free text based with codified information such as diagnosis codes and incorporating other sources of information such as laboratory data and procedural data to see if this adds value to detection and monitoring of infectious disease epidemics beyond what is currently possible through our current surveillance modalities.

### Future Work and Limitations

Other future work on CHESS to consider would include incorporating qualitative descriptions of severity. Such terms, either at the overall episode level, or in association with specific symptoms, could potentially add value to surveillance or even diagnosis of infectious (and possibly noninfectious) conditions. The current tool had components such as general condition (well, good, fair, poor, and alert) and appearance (toxic and nontoxic), but the primary care records available for analysis did not have sufficient data to allow us to validate this function. Validation would require implementing CHESS on a larger set of records and a more diverse set of free-text notes from primary care as well as emergency departments. Such expanded coverage would likely enhance our ability to discern signals from infectious disease outbreaks. It occurs to us that, having validated the algorithm at the level of phrases containing various symptoms, the work also sets the foundation for NLP tools to be used outside the confines of syndromic surveillance. For instance, new symptoms can easily be added to the dictionary to expand the application of the tool to noncommunicable disease–related conditions, to attempt what has been done using other systems, for instance, to classify clinical problem lists and detect postoperative complications [23,30]. However, such applications may need recognition of symptoms described by anatomy and be able to interpret other terms expressing uncertainty in the assertion status. This would require improvements to our current tool, including contextual learning modules that identifies terms based on where they are placed in the clinical record.

Furthermore, several limitations in our work should be acknowledged. First, we have described some of the weaknesses in CHESS’s algorithms from our qualitative review of those instances where misclassification occurred. We have already added keywords and misspellings identified in the validation set to the current version of CHESS, although a more generalizable way of dealing with misspellings would be ideal. Other improvements needed include an algorithm to identify different sentences within a single line of text (which we have since implemented) and a module to distinguish instances when the doctor advises the patient of future symptoms from currently reported symptoms (that we are now building). There were insufficient instances of these occurrences in primary care notes to allow us to validate these enhancements, but such advice upon discharge is likely to occur at much higher frequencies in emergency department as compared with primary care EMR.

Furthermore, it must be noted that although NHGP is a major primary care provider in Singapore and currently has the information systems to allow near-time access to their EMR for the NLP algorithm to be viably implemented in their context, it is unclear if the infrastructure for other primary care providers and emergency departments can support real-time surveillance. Singapore currently has a National Electronic Health Record system that receives contributions several times daily from various providers, including both primary care providers and emergency departments, and we are currently exploring the feasibility of using that as a platform to implement CHESS. However, in doing so, we must also recognize that CHESS was trained and validated only on NHGP notes. We expect additional shorthand forms, misspellings, and terminologies should our system be extended to other primary care systems, or to records from emergency departments (though preliminary testing of CHESS on a set of emergency department notes showed a good albeit slightly lower performance for our primary care notes with 93.2% precision and 86.3% recall). Even for use within NHGP, we acknowledge that the introduction of additional words and terms because of factors such as staff turnover or new methods of documentation may cause degradation of performance. Prospective implementation would hence require periodic revalidation, with retraining instituted should the performance drop below a satisfactory standard. These weaknesses are inherent in the keyword-based approach we adopted, where an exact match for a specific string of characters is required for detection by the parser; any new terms thus has to be manually added into the algorithm dictionary to be detected.

Currently there is a trend toward using automated approaches to NLP to identify new ontology and improve detection sensitivity, and this would be an alternative to manually adding keywords. However, there are several potential issues with such approaches. Topic modeling, for instance, requires large numbers of medical notes to come up with concept similarities within unstructured data. Although at a glance, this method of building the ontology may appear to be simple, it still needs to be verified and manually supervised. Moreover, in a study by Arnold et al [31], it was noted that the Latent Dirichlet allocation method of identifying topics resulted in lesser number of interpretable topics than a primary physician could identify. This was attributed to the fact that the Latent Dirichlet allocation method needs to identify topics from a highly specialized collection with a large vocabulary of related medical terminologies, which is not feasible without supervision. In another study, it was noted that the corpus for training NLP had almost 30% redundancy, where the doctor copies and pastes previous medical histories of a single patient. Redundancy can also occur when doctors at a hospital use a template for data entry (or in some cases for standard advice given to patients with a particular set of diagnoses). In such cases, as the NLP is trained using topic modeling, an inherent bias is created because of the increased probability of the co-occurrence of specific words [32]. Other automated NLP systems such as SimStat also require manual input to create an inclusion and exclusion dictionary from the list of words most frequently found and may thus also not be time-efficient [33]. Furthermore, clinical notes are full of spelling mistakes, abbreviations, and multi-word phrases, which makes it harder for automated NLP tools to identify patterns of occurrence. In this particular instance, the NHGP clinical notes were mostly short with an average of 4.7 words per phrase (maximum of 35 words per phrase), and each record had an average of 10 phrases. This was likely because of the high workload in the primary care setting, where clinicians had less time for more extensive documentation. Notes were hence to the point but rife with abbreviations and misspellings, and it is hence uncertain how an automated NLP technique might have performed. Therefore, although our method of manual coding to identify keywords was time-consuming, it proved to have sufficient performance, and we see it as a necessary step to serve as a benchmark algorithm for future work using automated NLP techniques. Moreover, the dictionary of terms used in local clinical practice that we compiled, though certainly not exhaustive, is an invaluable resource that can be exported into other systems to improve detection rates. For instance, IDEAL-X, an online machine-learning tool, requires a list of control vocabulary terms to improve on its statistical models of automated NLP [34].

### Conclusions

In conclusion, we have described the process of developing and validating CHESS, an NLP algorithm to extract information on signs and symptoms, along with information on assertion status and symptom duration from free-text primary care notes that we intend to make available for free download for researchers to access and build on. This simple rule-based concept extraction NLP tool could achieve good precision and recall approaching that for manual identification of symptoms and accurately identified most of the common infectious disease-related symptoms. Problems with performance were mainly because of the instances where we wanted to reduce false positives while improving sensitivity for a small proportion of situations where the documentation style was unusual or not found in our training dataset. Future steps would be to implement CHESS on a larger set of records and develop approaches to combine free text based with codified information such as diagnosis codes while comparing the outputs of such approaches with those from existing surveillance systems. There is also a need to test CHESS on a more diverse set of free-text notes from primary care as well as emergency departments, as expanded coverage would likely enhance our ability to discern signals from infectious disease outbreaks. We should also simultaneously test if newer approaches based on machine learning can serve as a more efficient and similarly effective way of updating our NLP algorithms.

## Appendix

Multimedia Appendix 1 Three supplementary tables, which include frequency of symptom in 1680 episodes, performance of CHESS algorithm on training dataset, and performance on validation dataset.

## Abbreviations

ANTLR: ANother Tool for Language Recognition
CHESS: Clinical History Extractor for Syndromic Surveillance
EMR: electronic medical record
ICD-10: International Statistical Classification of Diseases and Related Health Problems-10th revision
MCVS: Multi-Threaded Clinical Vocabulary Server
NHGP: National Healthcare Group Polyclinics
NLP: natural language processing
UMLS: United Medical Language System
