# Supplementary material for: Validation of a Natural Language Processing Algorithm for Detecting Infectious Disease Symptoms in Primary Care Electronic Medical Records in Singapore
Source: JMIR Med Inform. 2018 Jun 11;6(2):e36. doi: 10.2196/medinform.8204 (PMC6026305; doi:10.2196/medinform.8204)
Supplement: Multimedia Appendix 1 [file medinform_v6i2e36_app1.pdf]

Table E1. Frequency of symptoms detected by human coders within 1680 primary care records				
**Symptoms, sorted by frequency of affirmed cases in episode level**	Phrase Level (N = 8861)		Episode Level (N = 1680)	
	All instances	Affirmed (%)	All instances	Affirmed (%)
**Cough**	785	686 (87.4%)	744	646 (86.8%)
**Rhinorrhea**	667	518 (77.7%)	619	488 (78.8%)
**Sore throat**	710	447 (63.0%)	591	416 (70.4%)
**Fever**	1068	247 (23.1%)	928	228 (24.6%)
**Sputum**	261	214 (82.0%)	256	212 (82.8%)
**Skin lesions**	313	256 (81.8%)	183	154 (84.2%)
**Headache**	194	163 (84.0%)	172	142 (82.6%)
**Diarrhoea**	309	167 (54.0%)	271	137 (50.6%)
**Abdominal pain**	219	139 (63.5%)	202	128 (63.4%)
**Myalgia**	145	101 (69.7%)	143	99 (69.2%)
**Itch**	88	80 (90.9%)	82	76 (92.7%)
**Vomiting**	361	85 (23.5%)	347	75 (21.6%)
**Rash**	457	108 (23.6%)	345	72 (20.9%)
**Sensory Deficit**	260	85 (32.7%)	186	71 (38.2%)
**Dizziness**	134	77 (57.5%)	115	64 (55.7%)
**Chest pain**	182	64 (35.2%)	165	52 (31.5%)
**Nausea**	150	49 (32.7%)	149	49 (32.9%)
**Red eyes**	89	65 (73.0%)	53	36 (67.9%)
**Abdominal distension**	43	34 (79.1%)	39	34 (87.2%)
**Dyspnoea**	322	31 (9.6%)	286	31 (10.8%)
**Bleeding**	181	43 (23.8%)	148	31 (20.9%)
**Dysuria**	112	21 (18.8%)	108	20 (18.5%)
**Anorexia**	106	20 (18.9%)	103	20 (19.4%)
**Neck stiffness**	157	22 (14.0%)	146	19 (13.0%)
**Wheezing**	74	25 (33.8%)	65	19 (29.2%)
**Fatigue**	31	18 (58.1%)	30	18 (60.0%)
**Edema**	115	20 (17.4%)	106	15 (14.2%)
**Ulcer in mouth**	33	22 (66.7%)	21	14 (66.7%)
**Lymphadenopathy**	117	21 (17.9%)	104	11 (10.6%)
**Chills**	26	11 (42.3%)	26	11 (42.3%)
**Limb weakness**	318	12 (3.8%)	202	10 (5.0%)
**Weight loss**	60	12 (20.0%)	58	10 (17.2%)
**Hematuria**	27	14 (51.9%)	22	9 (40.9%)
**Jaundice**	127	9 (7.1%)	122	7 (5.7%)
**Altered state of consciousness**	424	10 (2.4%)	376	7 (1.9%)
**Epistaxis**	10	7 (70.0%)	7	5 (71.4%)
**Photophobia**	11	4 (36.4%)	11	4 (36.4%)
**Arthralgia**	5	4 (80.0%)	5	4 (80.0%)
**Orthopnea**	6	3 (50.0%)	6	3 (50.0%)
**Tachypnea**	12	3 (25.0%)	12	3 (25.0%)
**Haematemesis**	12	1 (8.3%)	12	1 (8.3%)
**Haemoptysis**	6	1 (16.7%)	6	1 (16.7%)
**Rebound tenderness**	51	1 (2.0%)	51	1 (2.0%)

**Seizure**	4	1 (25.0%)	4	1 (25.0%)
**Malena**	51	0 (0.0%)	48	0 (0.0%)
**Night Sweats**	4	0 (0.0%)	4	0 (0.0%)
**Cyanosis**	24	0 (0.0%)	24	0 (0.0%)

Table E2. Performance of NLP algorithm on the training set (n=840)						
	Phrase level			Episode level		
	Cases	Precision	Recall	Cases	Precision	Recall
**Respiratory**						
**Chest pain**	99	98.0%	99.0%	88	100.0%	98.9%
**Cough**	371	98.7%	99.7%	353	100.0%	99.7%
**Cyanosis**	12	100.0%	100.0%	12	100.0%	100.0%
**Dyspnoea**	163	98.2%	98.8%	147	98.6%	99.3%
**Orthopnea**	4	100.0%	100.0%	4	100.0%	100.0%
**Rhinorrhea**	308	98.7%	98.4%	286	99.0%	99.0%
**Sore throat**	349	96.9%	98.9%	280	97.9%	100.0%
**Sputum**	123	98.3%	96.7%	123	98.3%	96.7%
**Tachypnea**	9	100.0%	100.0%	9	100.0%	100.0%
**Wheezing**	35	97.2%	100.0%	31	100.0%	100.0%
**Gastrointestinal**						
**Abdominal Distension**	17	94.4%	100.0%	15	93.8%	100.0%
**Abdominal Pain**	104	91.0%	97.1%	98	94.1%	98.0%
**Diarrhoea**	131	100.0%	100.0%	115	100.0%	100.0%
**Nausea**	67	97.1%	98.5%	67	97.1%	98.5%
**Vomiting**	155	98.7%	98.7%	151	98.7%	98.7%
**Constitutional**						
**Anorexia**	55	94.8%	100.0%	54	98.2%	100.0%
**Chills**	16	100.0%	100.0%	16	100.0%	100.0%
**Fatigue**	17	45.9%	100.0%	17	47.2%	100.0%
**Fever**	496	98.6%	99.4%	439	99.8%	99.5%
**Myalgia**	76	89.2%	97.4%	75	91.3%	97.3%
**Weight loss**	34	87.5%	20.6%	34	87.5%	20.6%
**Others**						
**Altered state of consciousness**	207	96.3%	100.0%	187	98.9%	100.0%
**Arthralgia**	3	42.9%	100.0%	3	42.9%	100.0%
**Bleeding**	88	91.3%	71.6%	72	92.7%	70.8%
**Dizziness**	70	97.1%	97.1%	60	100.0%	100.0%
**Dysuria**	57	100.0%	93.0%	54	100.0%	92.6%
**Edema**	73	97.3%	98.6%	69	98.6%	98.6%
**Epistaxis**	8	100.0%	87.5%	6	100.0%	100.0%
**Haematemesis**	8	100.0%	75.0%	8	100.0%	75.0%
**Haemoptysis**	3	100.0%	33.3%	3	100.0%	33.3%
**Headache**	90	86.5%	100.0%	79	90.8%	100.0%
**Hematuria**	12	91.7%	91.7%	9	88.9%	88.9%
**Itch**	42	90.9%	95.2%	41	92.9%	95.1%
**Jaundice**	63	98.4%	98.4%	59	98.3%	100.0%
**Limb weakness**	166	96.7%	88.6%	104	97.0%	93.3%
**Lymphadenopathy**	59	98.1%	88.1%	49	97.8%	91.8%
**Malena**	31	100.0%	96.8%	28	100.0%	100.0%

**Neck Stiffness**	72	95.9%	97.2%	68	95.7%	98.5%
**Night Sweats**	0	NA	NA	0	NA	NA
**Photophobia**	8	100.0%	100.0%	8	100.0%	100.0%
**Rash**	228	98.7%	98.2%	173	100.0%	100.0%
**Rebound Tenderness**	21	100.0%	95.2%	21	100.0%	95.2%
**Red eyes**	39	80.6%	74.4%	23	95.0%	82.6%
**Seizure**	2	33.3%	100.0%	2	33.3%	100.0%
**Sensory deficit**	142	85.9%	94.4%	101	89.3%	99.0%
**Skin lesions**	131	73.6%	72.5%	85	76.8%	85.9%
**Ulcer in mouth**	19	100.0%	89.5%	12	100.0%	91.7%
**Overall**	4283	95.3%	95.6%	3738	96.7%	97.0%

Table E3. Performance of NLP algorithm on the validation set (n=840)						
	Phrase level			Episode level		
	Cases	Precision	Recall	Cases	Precision	Recall
**Respiratory**						
**Chest pain**	83	98.8%	96.4%	77	98.7%	97.4%
**Cough**	414	97.4%	99.8%	391	99.0%	99.7%
**Cyanosis**	12	100.0%	100.0%	12	100.0%	100.0%
**Dyspnoea**	159	98.7%	95.0%	139	99.3%	95.7%
**Orthopnea**	2	66.7%	100.0%	2	66.7%	100.0%
**Rhinorrhea**	360	99.4%	94.2%	333	99.4%	96.7%
**Sore throat**	361	96.2%	91.7%	311	98.0%	93.9%
**Sputum**	138	97.7%	92.0%	133	98.4%	92.5%
**Tachypnea**	3	100.0%	100.0%	3	100.0%	100.0%
**Wheezing**	39	100.0%	97.4%	34	100.0%	97.1%
**Gastrointestinal**						
**Abdominal Distension**	26	100.0%	96.2%	24	100.0%	100.0%
**Abdominal Pain**	115	93.1%	82.6%	104	94.8%	88.5%
**Diarrhoea**	178	98.1%	86.0%	156	98.5%	85.9%
**Nausea**	83	97.6%	96.4%	82	97.5%	96.3%
**Vomiting**	206	97.1%	96.1%	196	98.4%	96.4%
**Constitutional**						
**Anorexia**	51	91.1%	80.4%	49	90.7%	79.6%
**Chills**	10	90.9%	100.0%	10	90.9%	100.0%
**Fatigue**	14	42.4%	100.0%	13	43.3%	100.0%
**Fever**	572	98.8%	98.8%	489	99.8%	99.8%
**Myalgia**	69	88.2%	97.1%	68	88.0%	97.1%
**Weight loss**	26	100.0%	23.1%	24	100.0%	25.0%
**Others**						
**Altered state of consciousness**	217	98.1%	93.1%	189	98.9%	95.2%
**Arthralgia**	2	33.3%	100.0%	2	40.0%	100.0%
**Bleeding**	93	84.2%	51.6%	76	86.7%	51.3%
**Dizziness**	64	87.5%	87.5%	55	93.0%	96.4%
**Dysuria**	55	95.5%	76.4%	54	95.3%	75.9%
**Edema**	42	88.9%	95.2%	37	92.1%	94.6%
**Epistaxis**	2	100.0%	50.0%	1	100.0%	100.0%
**Haematemesis**	4	50.0%	25.0%	4	50.0%	25.0%
**Haemoptysis**	3	0.0%	0.0%	3	0.0%	0.0%
**Headache**	104	89.7%	92.3%	93	95.6%	92.5%
**Hematuria**	15	92.9%	86.7%	13	92.3%	92.3%
**Itch**	46	95.7%	97.8%	41	97.6%	97.6%
**Jaundice**	64	90.9%	93.8%	63	98.4%	96.8%
**Limb weakness**	152	95.0%	75.0%	98	94.0%	79.6%
**Lymphadenopathy**	58	94.1%	82.8%	55	94.0%	85.5%
**Malena**	20	86.4%	95.0%	20	95.0%	95.0%

**Neck Stiffness**	85	97.4%	89.4%	78	98.7%	94.9%
**Night Sweats**	4	100.0%	100.0%	4	100.0%	100.0%
**Photophobia**	3	100.0%	66.7%	3	100.0%	66.7%
**Rash**	229	97.0%	98.7%	172	98.8%	98.8%
**Rebound Tenderness**	30	100.0%	96.7%	30	100.0%	96.7%
**Red eyes**	50	66.0%	66.0%	30	80.0%	80.0%
**Seizure**	2	33.3%	50.0%	2	33.3%	50.0%
**Sensory deficit**	118	78.4%	73.7%	85	86.6%	83.5%
**Skin lesions**	182	73.6%	65.9%	98	71.2%	75.5%
**Ulcer in mouth**	14	88.9%	57.1%	9	87.5%	77.8%
**Overall**	4579	94.2%	90.0%	3965	96.0%	92.7%
